# Tire-road friction estimation and traction control strategy for motorized electric vehicle

**DOI:** 10.1371/journal.pone.0179526

**Published:** 2017-06-29

**Authors:** Li-Qiang Jin, Mingze Ling, Weiqiang Yue

**Affiliations:** State Key Laboratory of Automotive Simulation and Control, Jilin University, Changchun, China; Chongqing University, CHINA

## Abstract

In this paper, an optimal longitudinal slip ratio system for real-time identification of electric vehicle (EV) with motored wheels is proposed based on the adhesion between tire and road surface. First and foremost, the optimal longitudinal slip rate torque control can be identified in real time by calculating the derivative and slip rate of the adhesion coefficient. Secondly, the vehicle speed estimation method is also brought. Thirdly, an ideal vehicle simulation model is proposed to verify the algorithm with simulation, and we find that the slip ratio corresponds to the detection of the adhesion limit in real time. Finally, the proposed strategy is applied to traction control system (TCS). The results showed that the method can effectively identify the state of wheel and calculate the optimal slip ratio without wheel speed sensor; in the meantime, it can improve the accelerated stability of electric vehicle with traction control system (TCS).

## 1.Introduction

CONVENTIONAL internal combustion engine (ICE)-driven vehicles have incurred tremendous fossil fuel consumption, carbon footprint, and poisonous tailpipe emissions [1]. As far as possible to reduce carbon emissions on the one hand can improve the climate problem, on the other hand can also significantly impact the nature of emissions-optimal on-road power management [2]. So currently, it is imperative to explore the full carbon dioxide-saving potential for electric vehicles [3]. One of the major advantages of electric vehicles is the quick and precise torque response of the electric motor, which realizes a novel traction control system [4]. Torque control of electric motor via current gives the advantage of simplicity and fast response over the complicated torque control of an internal combustion engine which may depend on several parameters ranging from fuel valve angle to gas pedal position and several delay factors [5]. It is the developing trend of new generation electric vehicle driving systems.

Active safety technologies like Anti-Lock Braking System (ABS), Traction Control System (TCS) and Electronic Stability Control (ESC) have become hugely successful in recent decades. They have dramatically improved the traction and traffic ability, stability and safety of vehicles [6–8]. As for the control system, the ability to respond in real-time is a key requirement. Fuzzy-logic-based ABS/traction control could substantially improve longitudinal performance and offer significant potential for optimal control of driven wheels [6]. And the establishment of fuzzy rules and the selection of related parameters are very dependent on experience, it can reduce a lot of computation and has good robustness if parameters are chosen reasonably. PID control is widely used in control systems because of its clear structure and quick calculation, which make it all eligible for real-time control [9]. In in-wheel motor driven vehicles makes direct control of traction torque TCS possible, obviating the need to perform the colligation control of the power train, thus greatly simplified the control system structure. For instance, an anti-skid controller with PI regulator is designed and analyzed, based on back-EMF observer and dynamic model error observer [10]. The PID controller is widely employed in the industry, as well as in the TCS system. The PID control does not prevail now, however, compared with other strategies, it still has the value in engineering applications [11]. In this paper, the PID control is proposed into the TCS control of in-wheel motor driven vehicles.

One of the key points in the research field of automobile chassis control is to achieve the optimum slip rate and to identify the sliding state. Currently scholars have proposed theories and methods such as references [12–24]. A recursive least squares algorithm with forgetting was used to estimate the model parameters [18]. The model parameters have been optimized by executing the nonlinear least-squares algorithm, given large amounts of EIS test data [19]. The adaptive unscented Kalman filter (AUKF), fractional Kalman filter(FKF) and the extended Kalman Filter(EKF) is a dynamic system which is considered by Kalman filter, which is often used in target tracking system [20,21]. CHIA-SHANG LIU and HUEI PENG used modified adaptive observer and least square algorithm to estimate the road surface condition [14]. A Kalman filter is applied to a physical model of tire-road friction based on the wheel slip, using only standard sensors [12]. The optimal slip ratio is defined by the value corresponding to the peak friction factor between the road and tire. It is also the key technology for estimating road adhesion in real time for vehicle chassis control systems [4, 22]. So far, the technology, especially the identification method of optimum slip ratio, has some room for improvement in terms of accuracy. Currently, the general method uses existing data which is gained by checking tables, and is difficult to adapt to the complexity of the roads [3, 6–7]. Li, et al, made the torque increase or decrease to control the wheel slip ratio in idea scope [22].

In recent years, many theoretical studies based on tire-road friction estimation are conducted by scholars. Firstly, it is commonly assumed that the tire friction and the slip ratio have a linear relationship at low slip ratios, the slip state can be calculated using the slope change of the friction vs slip ratio curve generated by a Kalman filter. Secondly, the slip state can be calculated using the slip ratio and the adhesion coefficient; the slip ratio is obtained by calculating vehicle speed and rotational velocity, and the adhesion coefficient is predicted using the extended Kalman filter (EKF). Thirdly, based on global position system (GPS), the sliding state can be calculated by the lateral acceleration, yaw velocity (obtained by gyroscope) and the lateral adhesion coefficient. The lateral adhesion coefficient is obtained by identifying the steering angle. This approach relies on the ability of GPS to display coordinates precisely. Finally, the slope of the curve between friction coefficient and slip ratio is defined as the normalized brake stiffness. According to tire friction characteristics, tires have the maximum braking force when the normalized brake stiffness is zero. The Euler approximation theory and the least square algorithm are used to determine the generalized braking stiffness of the zero position, which is used to determine the sliding state. These methods are difficult to apply to the real car for several reasons including timeliness, accuracy of recognition and cost. At present, these methods are appropriate for specific areas, or are still at the stage of theoretical research, lack of real vehicle test to verify it. Li *et al*. proposed a comprehensive tire-road friction coefficient estimation method which is based on signal fusion method under complex maneuvering operations inclusive of braking, driving and steering. This method is relatively timely and accurate to satisfy the control demands [22].

According to the actual state of the electric wheel, an ideal model for the inertia of the non-slip wheel is established based on the tire-road friction estimation method. The model’s self-motion adaptation method is used to identify the difference inertial parameters between the the actual electric-driving wheels and the ideal model, which determines the sliding state of the wheel. This method works well in certain conditions, but because of a wide variety of vehicles and roads, it is not suitable for all driving conditions [24]. Velimir C´ irovic proposed a new approach for improving of the longitudinal wheel slip control based on dynamic neural networks. This approach is based on dynamic adaptation of the brake actuation pressure, during a braking cycle, according to the identified maximum adhesion coefficient between the wheel and road [25].

From the above discussion, it is urgent demand to detect tire-road friction coefficient in vehicle dynamics and control. This paper advances a theory which is based on real-time sensor signals of speed and driving torque of motorized wheels to identify the electric-driving wheel slip ratio and the optimum slip ratio, and proposes a traction control strategy which is implemented based on the theory. At the same time, we also consider the algorithm is highly efficient in solution and calculation, and can be used in establishing real-time control system. Therefore, the approach is simple and dynamic-response is quick.

## 2. Estimation of optimum slip ratio on driving roads in real-time

### 2.1 Estimation of road adhesion for electric vehicle with motorized wheels

**Fig 1:**
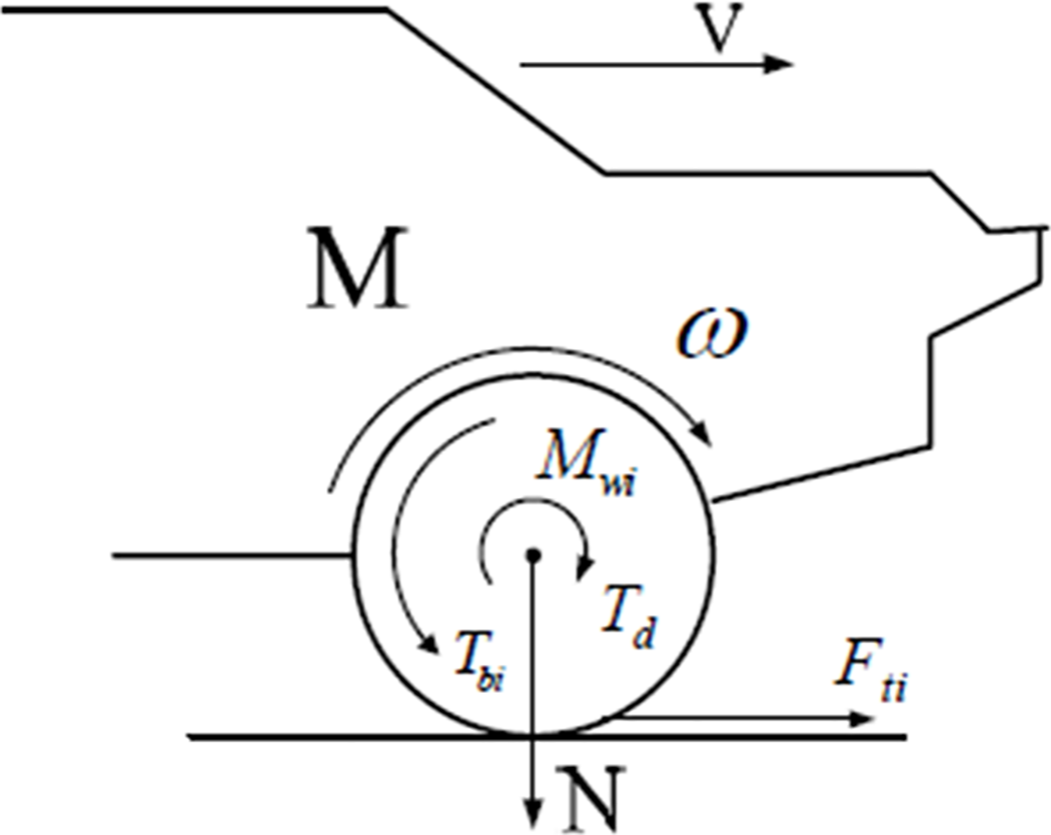
Quarter model of vehicle.

In order to solve the difference problem, the electric vehicle drive motor adopts torque control. The one quarter vehicle model is shown in Fig 1, the wheel and body dynamics equation can be expressed:

$$I_{w}\frac{dw}{dt}=T_{m}-F_{d}.r$$ (1)

$$M\frac{dV}{dt}=F_{d}$$ (2)

$$u(s)=\frac{F_{d}}{N}$$ (3)

Where *T*_*m*_ is the torque transmitted from the motor (N∙m); *F*_*d*_ is the friction between the road and the tire (N); *I*_*w*_ is the moment of inertia of the wheel (kg∙m^2^); *M* is the sprung mass acting on a single wheel (kg); *w* is the rotation speed of the wheels (rad/s); *V* is the body velocity (m/s); *F*_*d*_ is the traction; and the normalization traction can be shown to be:

$$u(s)=\frac{1}{Nr}\left(T_{m}-I_{w}\frac{dw}{dt}\right)$$ (4)

Where *u* is the adhesion coefficient between tire and road surface which is a function of wheel slip rate. The friction characteristics can be calculated using the wheel torque and rotational speed. The formula is also applying to conventional vehicles, but it is difficult to obtain the accurate driving torque of each wheel in real-time due to the traditional automotive transmission system, and it is difficult to estimate the friction characteristics based on Eq 4. For the electric vehicle driving by in-wheel motor, the torque and velocity of the wheel can be easily obtained. Therefore, the Eq (4) can be used to calculate the road friction characteristics.

### 2.2 Estimation strategy for optimum slip ratio in real-time

**Fig 2:**
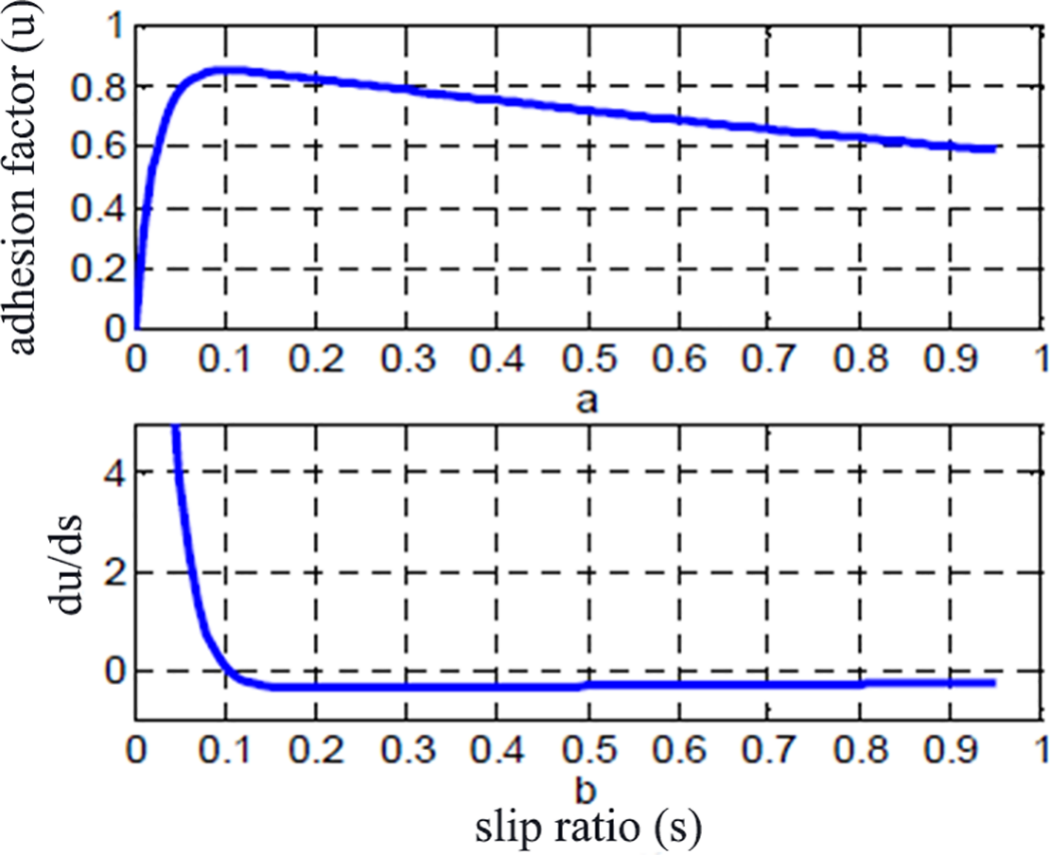
Relations for adhesion coefficient and slip ratio:(a) adhesion factor with slip ratio; (b)du/ds with slip ratio.

Fig 2 shows the characteristic curve of the road slip. Where *s*_0_ is the slip rate corresponding to the maximum adhesion factor between the road and the tire *u*_max_. Fig 2(B) is the characteristic curve of the derivative of the road adhesion and slip rate *du*/*ds*. It can be seen that when *s* < *s*_0_, then *du*/*ds* >0. At this stage, the adhesion coefficient increases as the slip rate increases, the wheel does not slip and the wheel are in a steady state. When *s* > *s*_0_, then *du*/*ds*<0. At this stage, the adhesion coefficient decreases as the slip rate increases, the wheel begins to slip and the wheel is in an unstable state.

When the vehicle is running, the variation of the adhesion coefficient and the slip rate between the tire and the road surface has a time course, thus:

$$\frac{du(s)}{ds}=\frac{du}{dt}.\frac{dt}{ds}$$ (5)

The sign of $\frac{du(s)}{ds}$ can be shown by $\frac{du}{dt}.\frac{ds}{dt}$. According to the characteristics of the adhesion coefficient and the slip ratio, the following rules can be obtained.

For accelerating wheels:

1. When the wheel is running in the non-stable state, this time *s* > *s*_0_, the adhesion coefficient decreases with time and slip ratio increases with time. Then:
  $$\frac{du}{dt}<0,\;\frac{ds}{dt}>0$$ (6)
2. When the wheel is running in a stable state, this time *s* ≤ *s*_0_, both the adhesion coefficient and slip ratio increase with time.
  $$\frac{du}{dt}\geq 0,\;\frac{ds}{dt}>0$$ (7)

For decelerating wheels:

1. When the wheel is running in a non-stable state, this time *s* > *s*_0_, the adhesion coefficient increases with time and the slip ratio decreases with time. Then:
  $$\frac{du}{dt}>0,\;\frac{ds}{dt}<0$$ (8)
2. When the wheel is running in a stable state, this time *s* ≤ *s*_0_, both the adhesion coefficient and slip ratio increase with time.
  $$\frac{du}{dt}\leq 0,\;\frac{ds}{dt}<0$$ (9)

Based on the above analysis, when the wheel is running from the stable state to the non-stable state, the wheel begins to slide ($\frac{du}{dt}$<0). $\frac{du}{dt}.\frac{ds}{dt}$>0 determines that the wheel runs from an unstable state to a stable state. Thus:

$$\frac{du}{dt}=\frac{d\left(\frac{1}{Nr}\left(T_{m}-I_{w}\frac{dw}{dt}\right)\right)}{dt}$$ (10)

For the road adhesion coefficient, the vertical load on the wheel and the wheel radius change little. Therefore, the vertical load of the wheel can be regarded as a constant, hence

$$\frac{du}{dt}=\frac{1}{Nr}\frac{d\left(T_{m}-I_{w}\frac{dw}{dt}\right)}{dt}$$ (11)

For in-wheel motor driving, it is easy to accurately estimate the torque *T*_*m*_ and the wheel rotation speed in real time. By detecting the change of $\frac{d\left(T_{m}-I_{w}\frac{dw}{dt}\right)}{dt}$, the wheel slip state can be identified. In order to conveniently express, the following equation is presented.

$$\varepsilon =\frac{d\left(T_{m}-I_{w}\frac{dw}{dt}\right)}{dt}$$ (12)

$$\sigma =\frac{ds}{dt}$$ (13)

In practical application, the wheel skidding state can be estimated by judging the positive and negative of the two cycles of *ε*, namely *ε*(*k*−1) and *ε*(*k*). In the accelerating process, if *ε*(*k*−1) >0 and *ε*(*k*) <0, the wheel begins to slip. The slip ratio on this point is the optimum slip ratio *S*_0_. Hence, the real-time calculation of peak adhesion coefficient can be achieved. In the decelerating process, if *ε* × *σ* >0, the wheel stops slipping. The estimation method is achieved by using the dynamic parameters of the wheel.

**Fig 3:**
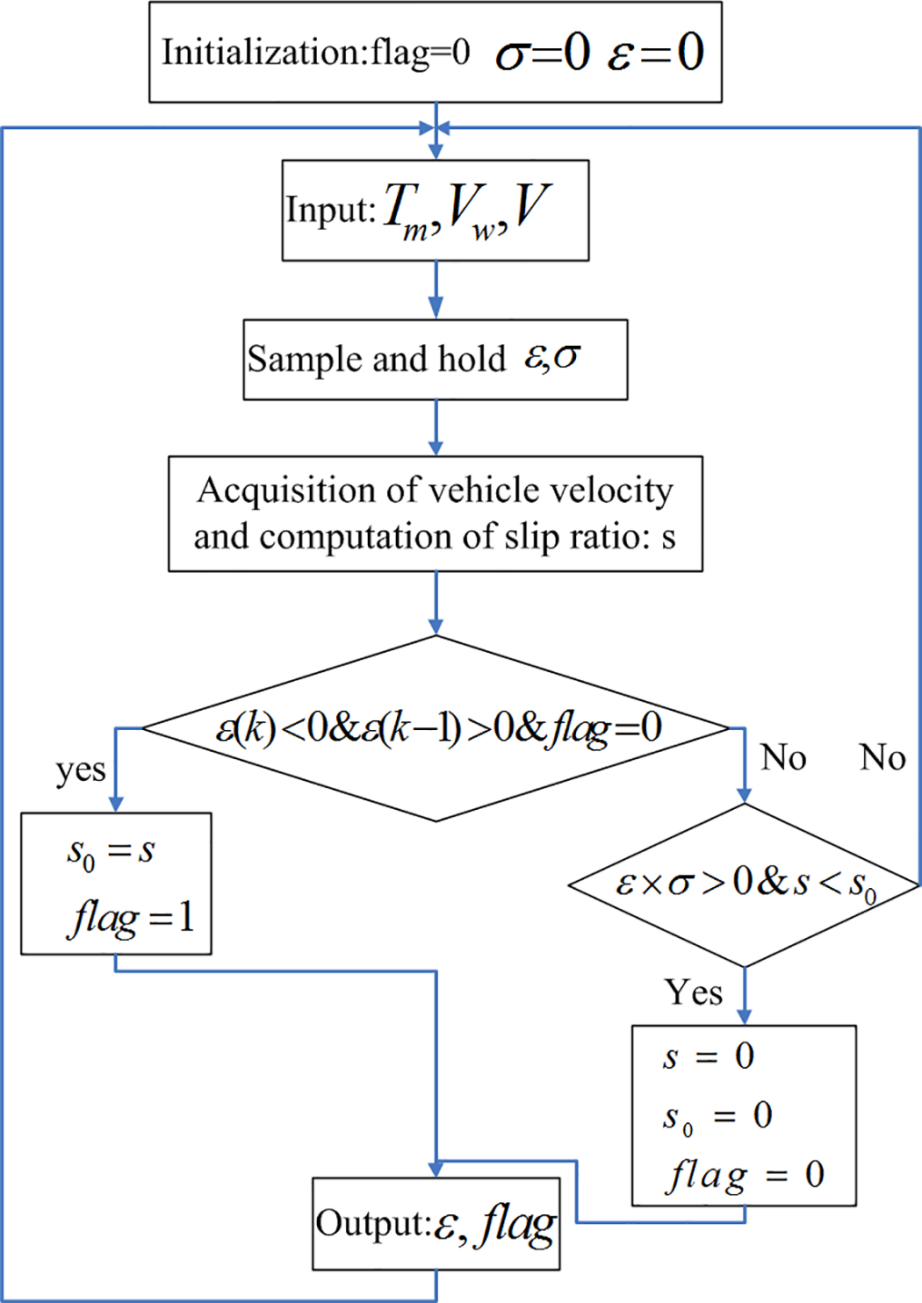
Estimation logic for optimal slip ratio.

The flow chart of the estimation method is shown in Fig 3. *flag* is the indicator of wheel slippage, which is used to determine whether the wheel is running in skidding state.

### 2.3 Vehicle velocity estimation

It is difficult to obtain the accurate speed when using the conventional powertrain to control TCS. According to the method proposed in this paper, it is found that the wheel slip of the electric wheel drive vehicle can be monitored in real time. Therefore, the speed calculation becomes easier. For a straight-driving car, if you do not consider the road roughness caused by the vertical component of the wheel speed, the wheel speed of each wheel is equal to equal speed. While steering, the kinematic relationship between the horizontal component of each wheel's center speed and vehicle speed is as follows:

$$\begin{array}{c} \nu_{h\omega 1}=\left(u+\frac{1}{2}B\gamma -l_{f}q\sin\left|\theta \right|\right)\cos\delta_{f}+\left(\nu +\alpha \gamma \right)\sin\delta_{f}\\ \nu_{h\omega 2}=\left(u+\frac{1}{2}B\gamma +l_{f}q\sin\left|\theta \right|\right)\\ \nu_{h\omega 3}=\left(u-\frac{1}{2}B\gamma -l_{f}q\sin\left|\theta \right|\right)\cos\delta_{f}+\left(\nu +\alpha \gamma \right)\sin\delta_{f}\\ \nu_{h\omega 4}=\left(u-\frac{1}{2}B\gamma +l_{f}q\sin\left|\theta \right|\right) \end{array}\}$$ (14)

u=Vcosβν=Vsinβ (15)

It ignores the pitching motion factor and considering the small angle relationship cos *β* = 1, sin *β* = 0. The expression above can be simplified as:

$$\begin{array}{c} \nu_{h\omega 1}=\left(V+\frac{1}{2}B\gamma \right)\cos\delta_{f}+\left(\alpha \gamma \right)\sin\delta_{f}\\ \nu_{h\omega 2}=\left(V+\frac{1}{2}B\gamma \right)\\ \nu_{h\omega 3}=\left(V-\frac{1}{2}B\gamma \right)\cos\delta_{f}+\left(\alpha \gamma \right)\sin\delta_{f}\\ \nu_{h\omega 4}=\left(V-\frac{1}{2}B\gamma \right) \end{array}\}$$ (16)

In Eq (16), the wheel speed can be expressed as a function of speed and steering angle. Steering Angle can be achieved by the steering wheel Angle sensor or steering system ECU, while the vehicle speed can be calculated by the product of the wheel speed and the wheel radius. In this article, we mainly discuss whether the electric driving wheel slip can be monitored and the optimal slip ratio recognition theory, we can calculate the vehicle speed via the none-slipping wheel, ignoring the slipping wheels.

The principles are:

□ During the straight driving, the vehicle speed is the non-skid minimum wheel velocity. If all the wheels are sliding, the system reduce the four-wheel drive torque. Meanwhile, the minimum speed of the four wheels is continuously detected and as the speed signal slip rate is calculated until there is a wheel back to the non-skid state. Then, non-skid wheel speed is used to calculate the speed to control the other wheels.
□ During the turning driving, the vehicle speed can be estimated by rotation difference, steering angle in Eq (16).

### 2.4 Control system structure of vehicles driven by in-wheel motors

**Fig 4:**
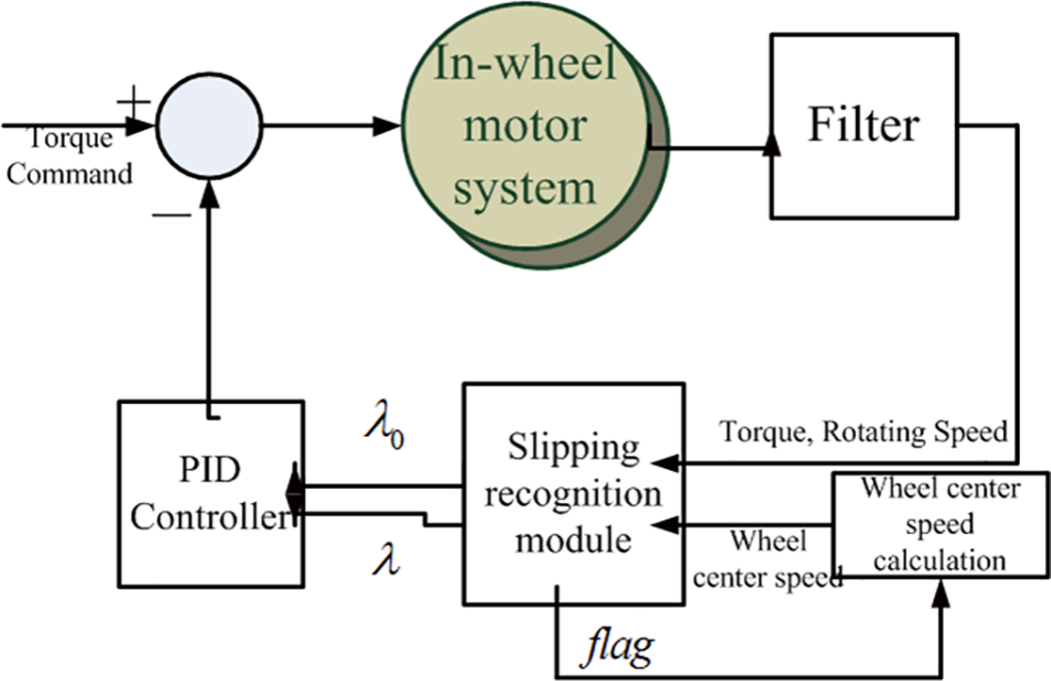
TCS structure of in-wheel motor driven system.

The TCS structure of the in-wheel motor driven system is showed in Fig 4. The system consisted of the filter module, slipping recognition module, vehicle speed calculating module and the PID control module. According to the input motor torque, rotating speed and wheel center speed, the slipping recognition module can identify whether the wheel slips and gives the sign *flag*, the slip rate *λ* and the corresponding slip rate *λ*_*o*_ of the maximum adhesion coefficient. The slipping rate recognition module has to carry out the derivative calculation on the motor torque and the motor rotating speed. However, the real output motor torque and rotating speed have high frequency noise. To avoid the high frequency oscillation after derivation, the low pass filter is designed for the output signal processing from the motors. The control of traction torque is implemented by adjusting the input torque command of the control system.

The wheel center speed module determines the sliding status based on the flag value, and then calculates the speed of each wheel according to the speed of the non-sliding wheel.

According to the error between λ_0_ and λ, the PID controller provides the negative feedback to the torque command. When the slipping recognition module recognizes that there is no slipping, the outputs of λ_0_ and λ are 0, thus the PID controller is in idle status.

## 3. Typical simulation experiments and results

### 3.1 verify the accuracy of the model

For the validation, the dynamics model with 18 freedoms for electric vehicle with motorized wheels is made, inclusive of the six freedoms of the vehicle body and the twelve freedoms for the rotation, steering and vertical motion of the four wheels. The “Magic formula” is used in the tire model and the driving motor is induction motor. Based on the model, the simulation desktop of the electric vehicle with motorized wheels is build using MATLAB/Simulink. With high precision and less parameters, the desktop can simulate many kinds driving mode of vehicle. The simulation validation of road adhesion estimation method is carried out by this model. Parameters of the test vehicles are shown in Table 1. The maximum power of the motor is 25kW and maximum torque is 400N⋅m, as shown in Table 2.

**Table 1:** Vehicle specifications for simulation.

Total Mass	1483 kg	Wheelbase	2.662 m
Wheel Inertia	3.11 kg.m^2^	Tread	1.44 m
Wheel Mass	38 kg	Wheel Radius	0.285 m

**Table 2:** Main specifications for driving motor.

Rated Power kw	17	Max Power kw	25
Rated Torque N∙m	180	Max Torque N∙m	400

**Fig 5:**
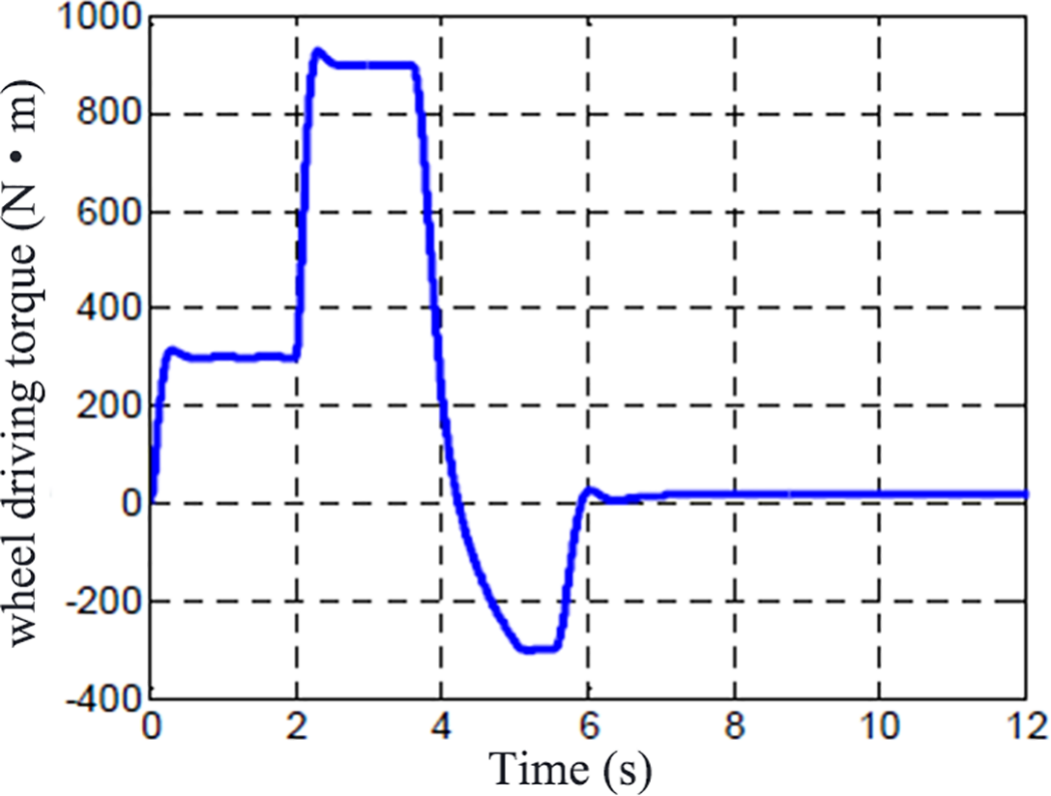
Wheel driving torque.

**Fig 6:**
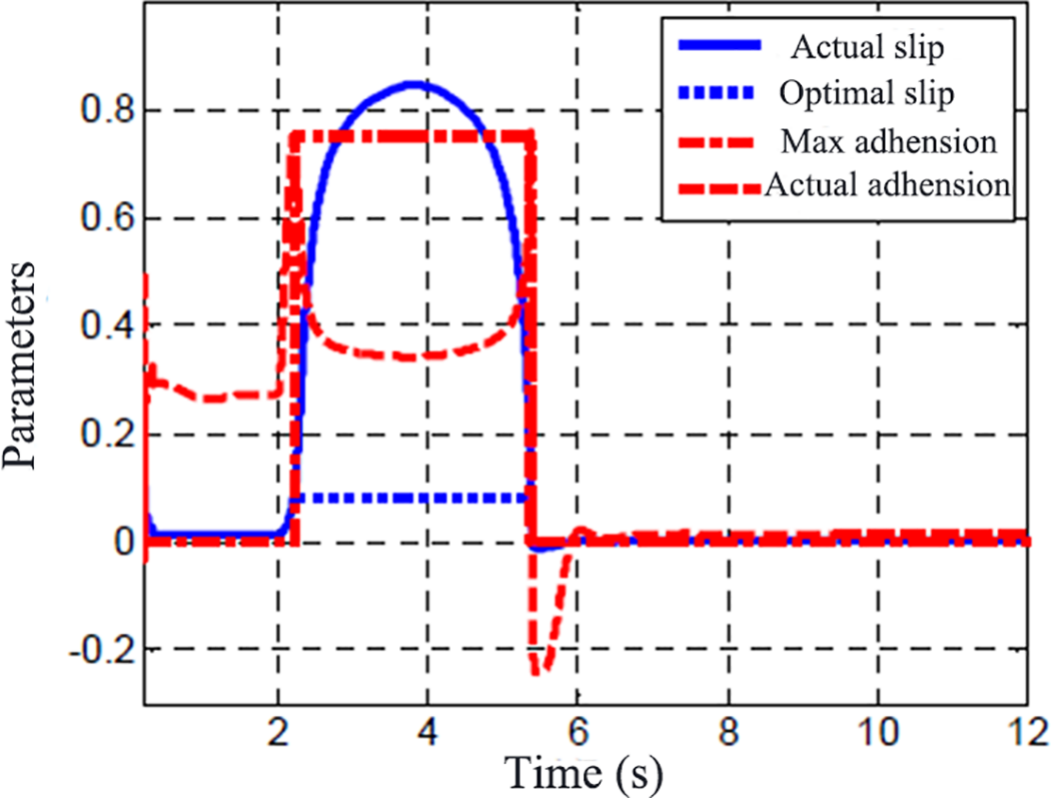
Output by road identifier.

The vehicle running at 50 km/h and after 2 s, vehicle began to accelerate quickly. The driving torque of the wheel is shown in Fig 5 and the road adhesion/slip ratios are presented in Fig 6. The vertical ordinate in the Fig 6 is the curve of the road surface adhesion coefficient (u), the optimal slip ratio (λ_0_) and the maximum adhesion coefficient (u_max_). The simulation show that the detector has the ability to capture the tire and road adhesion characteristics of the inflection point.

The optimal slip ratio (λ_0_), the max road adhesion(u_max_), the real slip ratio (λ) and road adhesion (u) are got by road adhesion identifier which is designed based on the presented estimation method. It can be seen that road friction factor increases to peak value quickly and then decreases quickly because of the wheel skidding. After 6s, the road friction increases quickly because the driving torque decreases and wheel slip ratio decrease too. The road friction factor increases to a peak value when the slip ratio is an optimal slip ratio. The optimal slip ratio and maximum road adhesion are accurately calculated as well as the wheel skidding state.

### 3.2 Simulations of acceleration under low adhesion road

**Fig 7:**
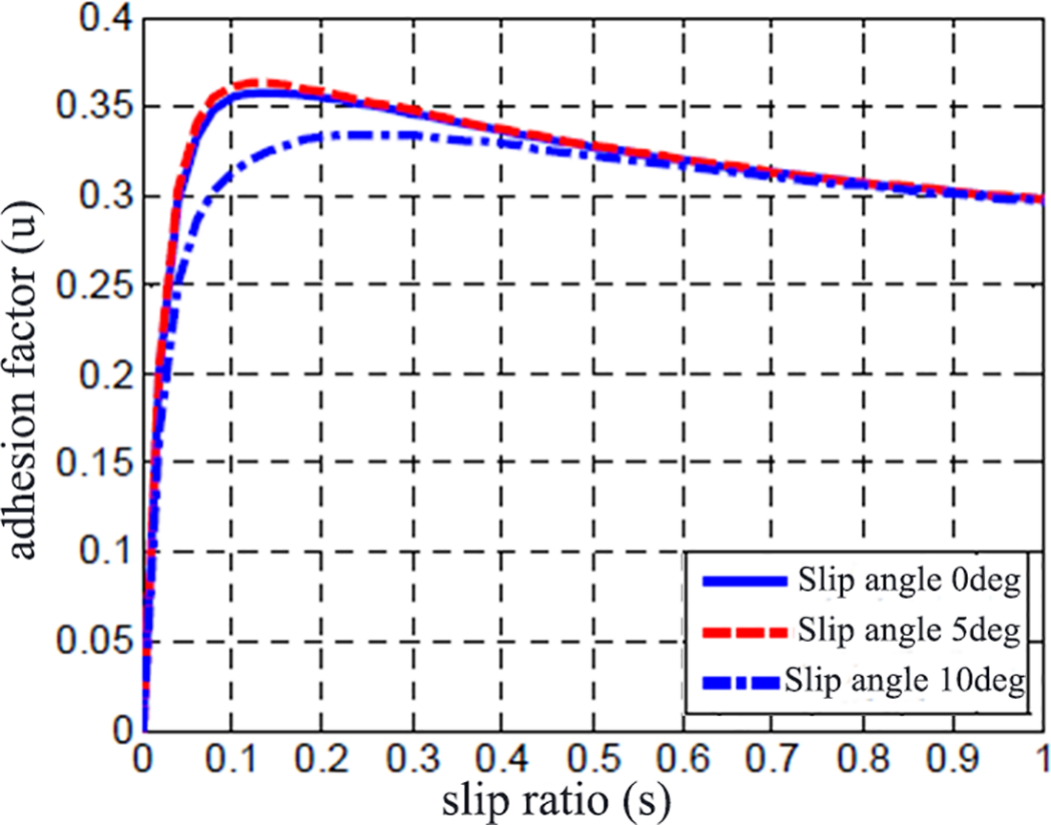
Adhesion characteristics of Road I.

**Fig 8:**
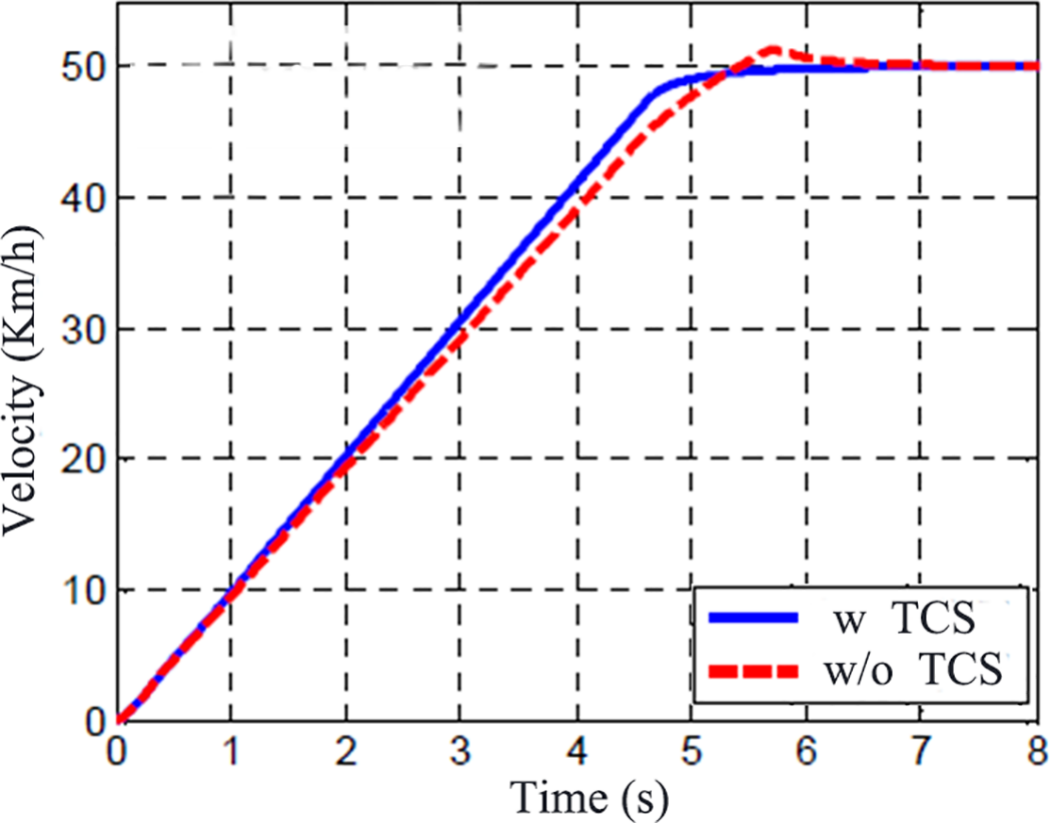
Vehicle speed variation.

The road adhesion characteristic is shown in Fig 7 while Fig 8 shows the vehicle speed for the TCS and no TCS. It clearly shows that the vehicle’s acceleration ability is enhanced obviously and the vehicle can accelerate to the target speed smoothly under the action of TCS.

**Fig 9:**
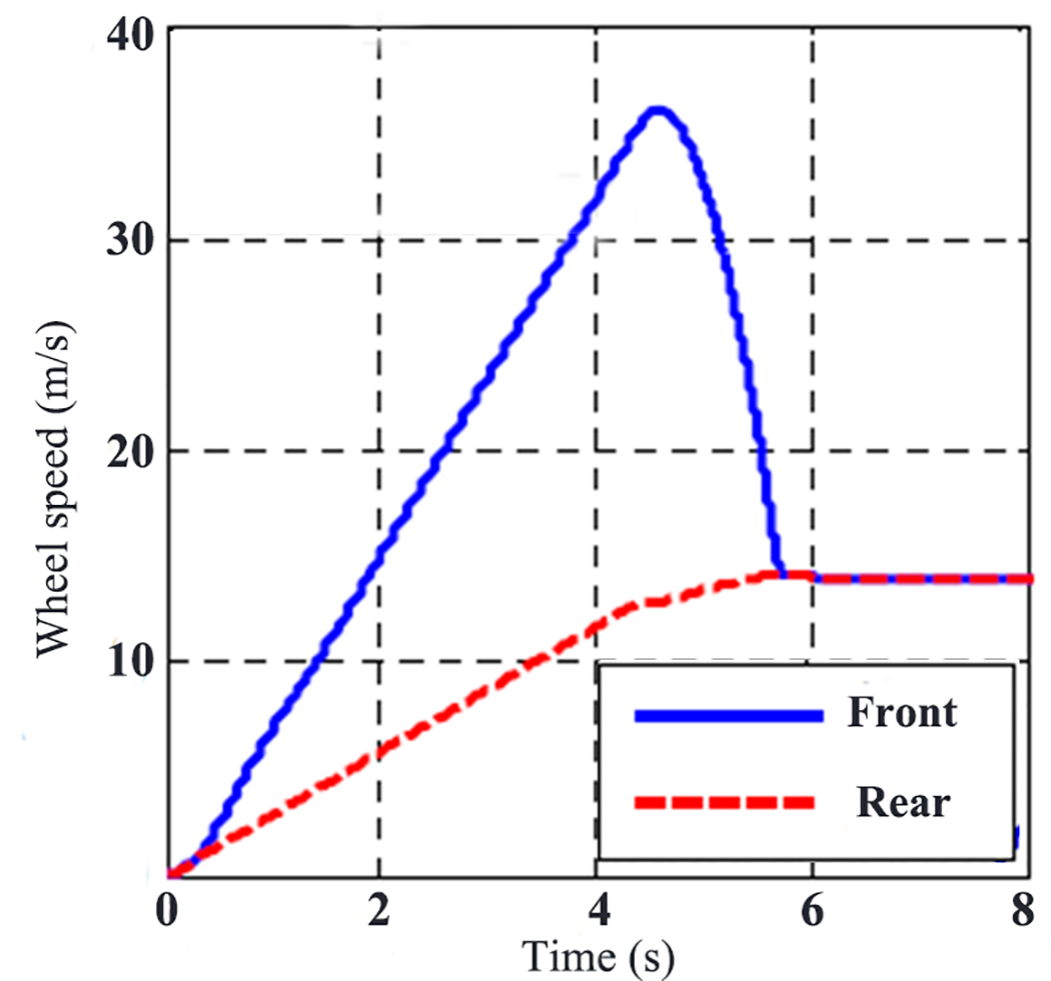
Wheel rotating speed without TCS control.

**Fig 10:**
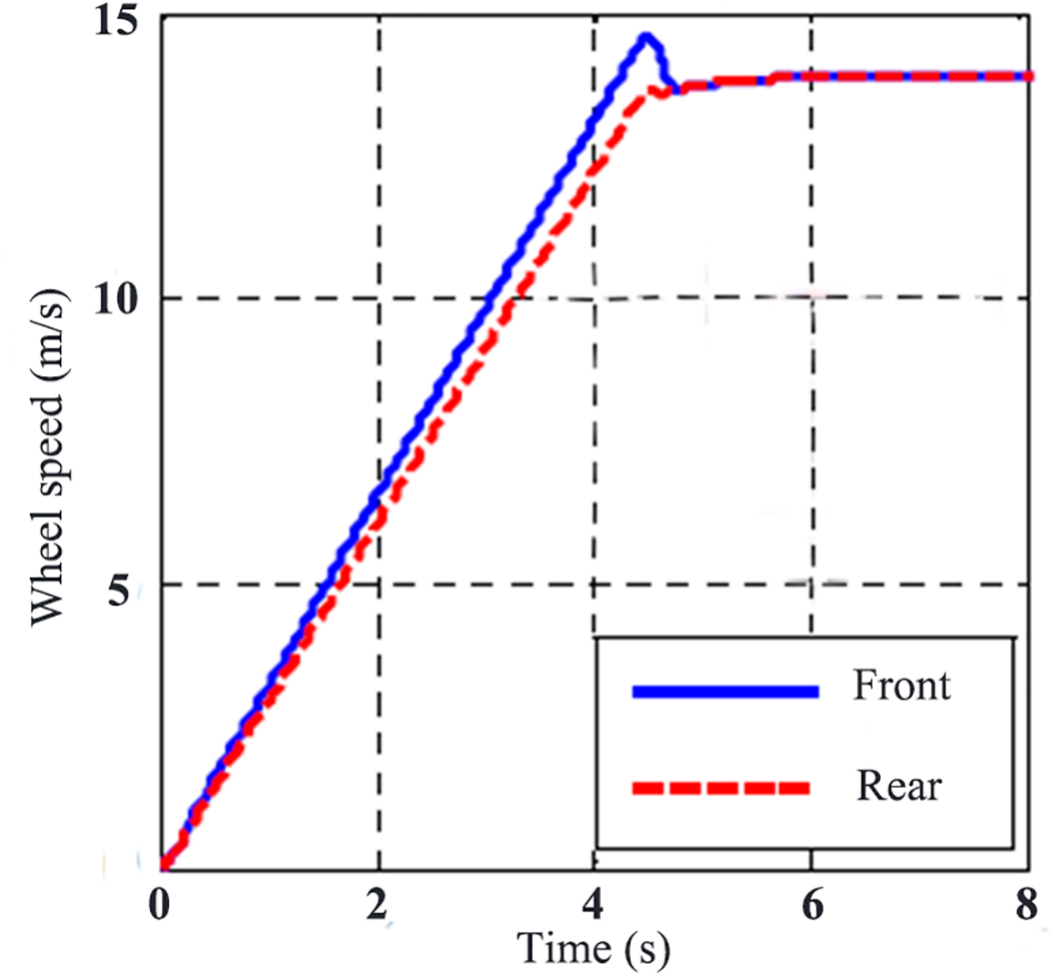
Wheel rotating speed with TCS control.

Fig 9 is the wheel rotating speed without TCS control. From it, there is no slipping on rear wheel during the whole acceleration process, while the front wheel accelerates sharply to the maximum slip rate in the end. Fig 10 is the wheel rotating speed with TCS control. Under the control of TCS, the slip rate can be maintained under a certain value to get the maximum traction.

**Fig 11:**
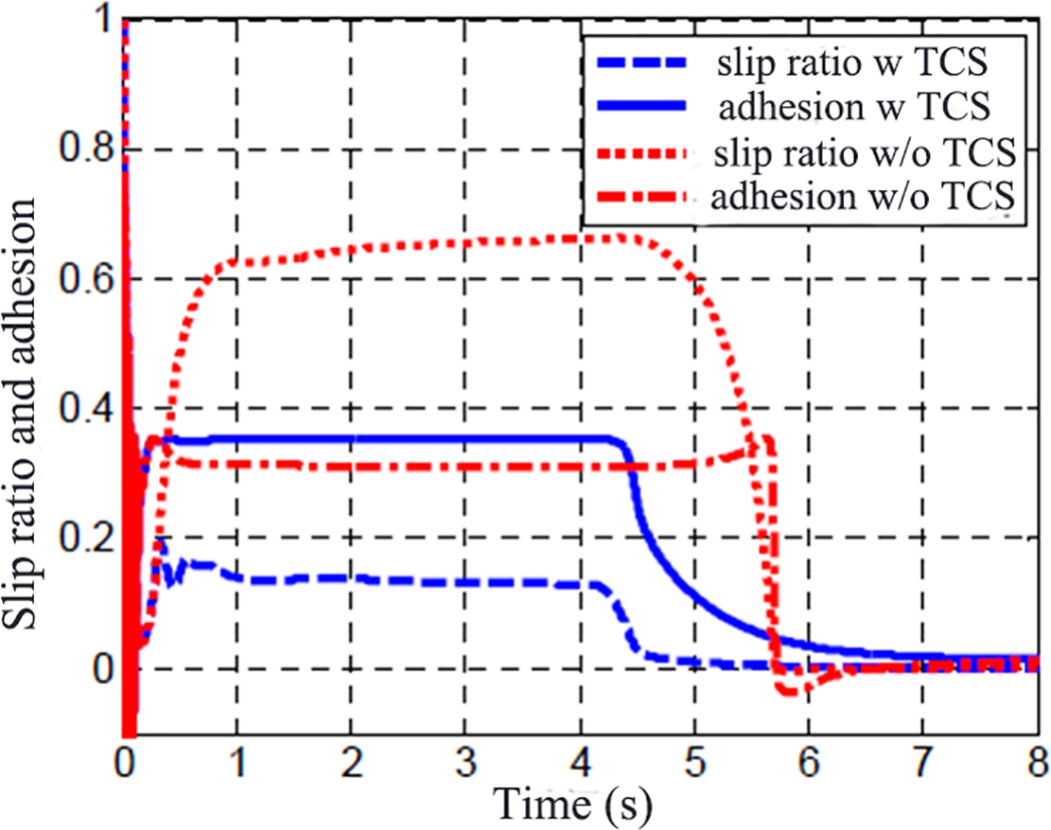
Comparison of adhesion coefficient and slipping rate.

**Fig 12:**
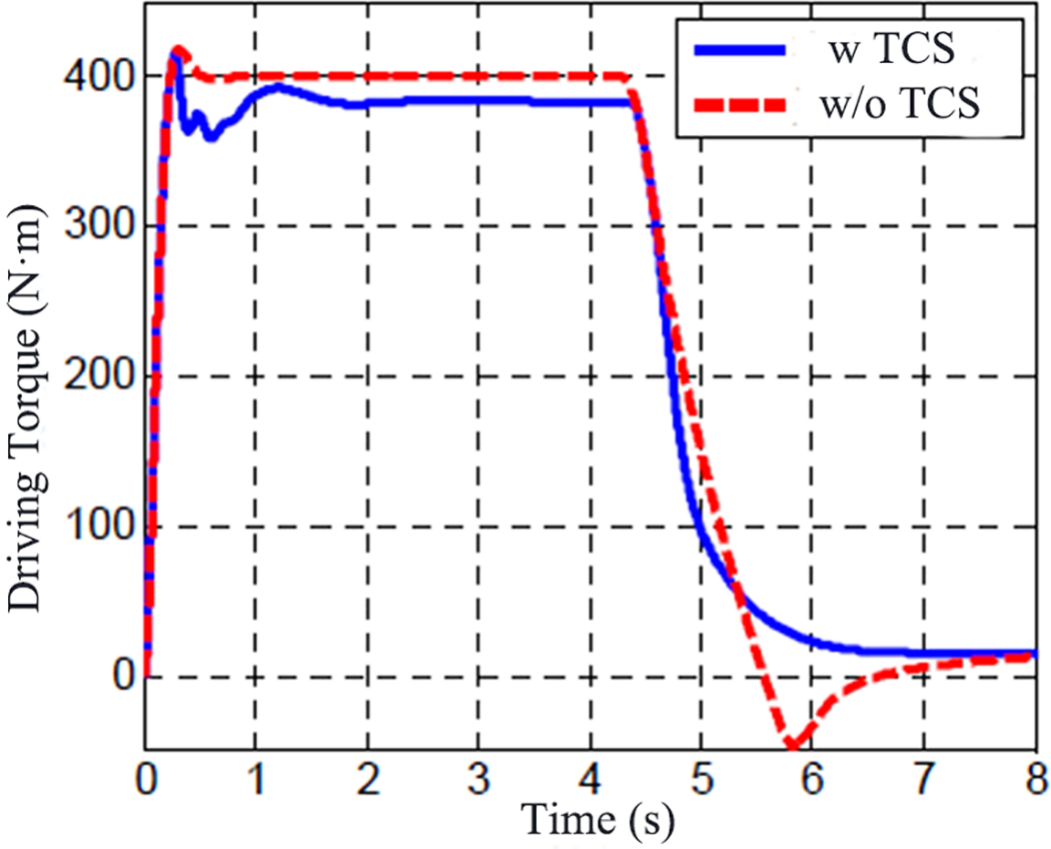
Comparison of traction variation.

Fig 11 displays the comparison of road adhesion coefficient and slip rate with and without TCS control. Without TCS control, the slip rate is close to 0.7, while the adhesion coefficient is obviously smaller than the maximum adhesion coefficient. With the effect of TCS, the wheel slip rate can be maintained around the optimum slip rate, which is shown in Fig 7. While the road adhesion coefficient keeps the maximum value to take the full advantage of the capability of road adhesion. Fig 12 is the wheel traction variation with and without TCS control. When accelerating, there is no big difference between the two controllers. However, the differential value takes the wheel slip rate beyond the slip rate under relative maximum road adhesion, thus taking the wheel movement into the unstable area, and results in over-slipping of the wheel.

## 4. Road test

**Fig 13:**
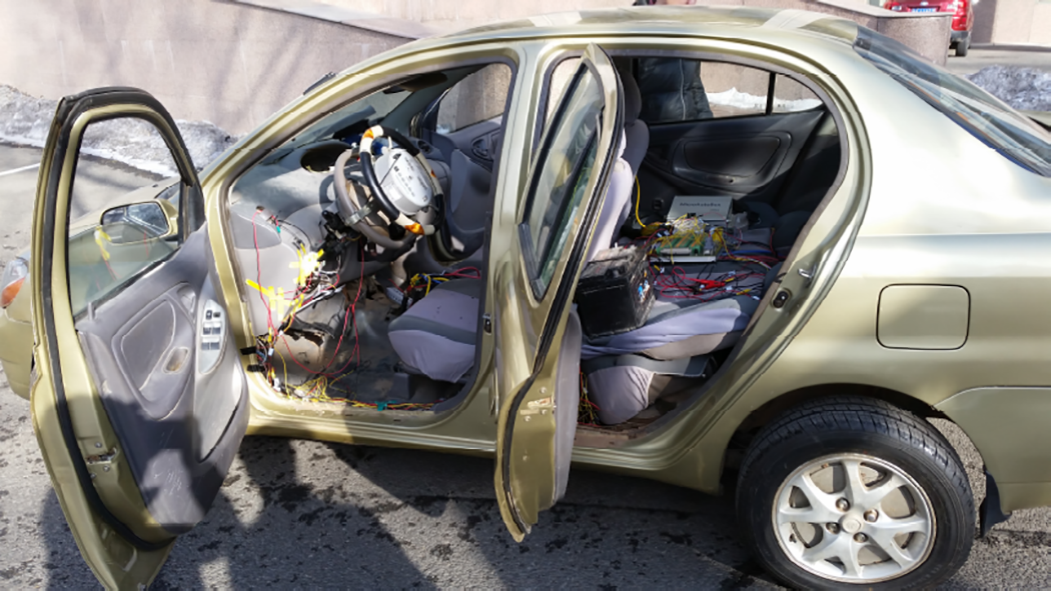
The tested vehicle.

To verify the control algorithm, the vehicle tests were performed on the campus of Jilin University. The electric vehicle(EV) with electric motored wheels is modified on the basis of the gas engine, make full use of the original car parts including the body, suspension system, brakes, etc. At the same time, remove the original vehicle power system, fuel tank and other parts, make more space for 4 wheel motor and its controller, the power batteries, vehicle powertrain control (VCU) and strong electric control box(including DC-DC, Power electric relay). The tested car is shown in Fig 13.

**Fig 14:**
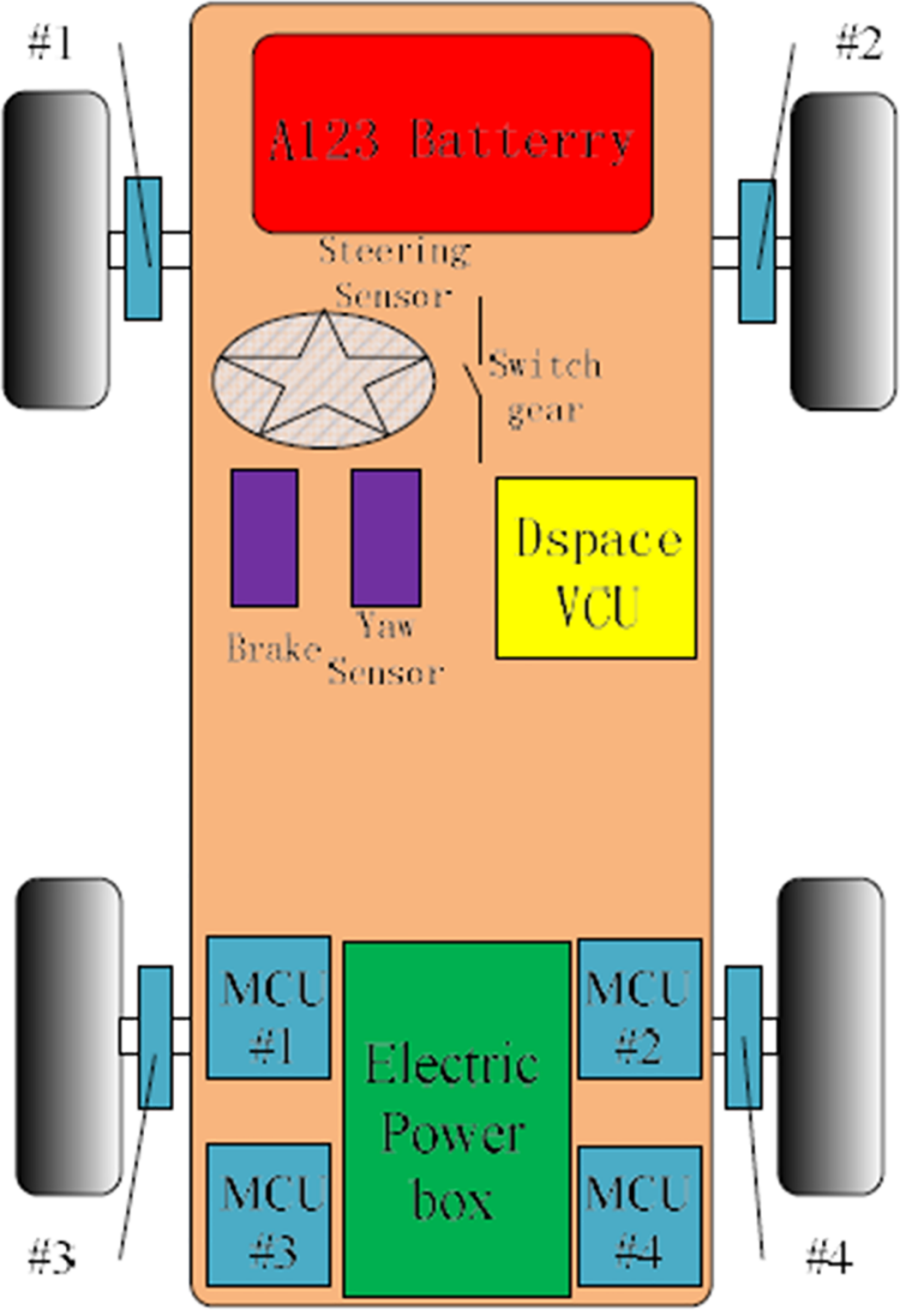
The overall vehicle arrangement.

The battery placed in the engine nacelle, four-wheel motor is respectively embedded in the four wheels, four electric motor controllers are respectively arranged on both sides of the box with fixed in the luggage compartment. The VCU uses Micro-autobox Dspace, which is placed in the back seat. Simultaneously, we installed the wheel speed sensor, the dual axis acceleration sensor and the yaw rate sensor. The Automobile tires were made of snow tires. The tests were demonstrated on icy road with maneuvering operations for TCS runs. The test items were carried out under the condition of full throttle and light load. As shown in Fig 14.

**Fig 15:**
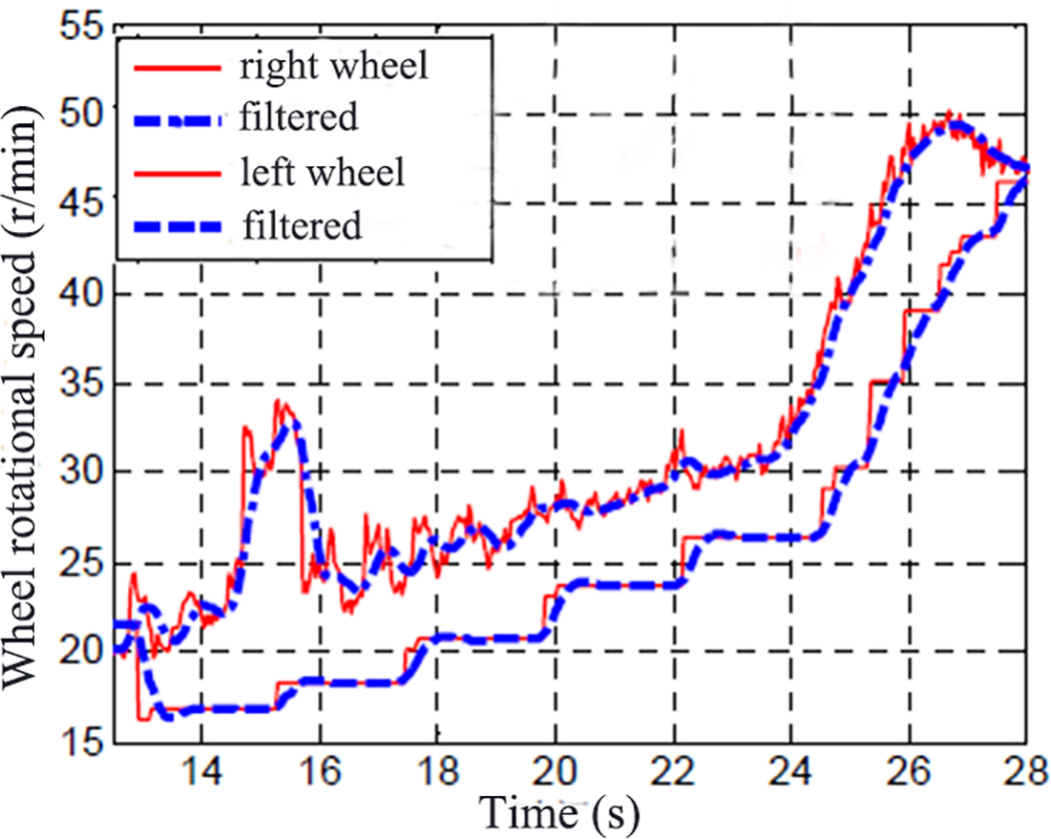
Original wheel speed and filtered.

**Fig 16:**
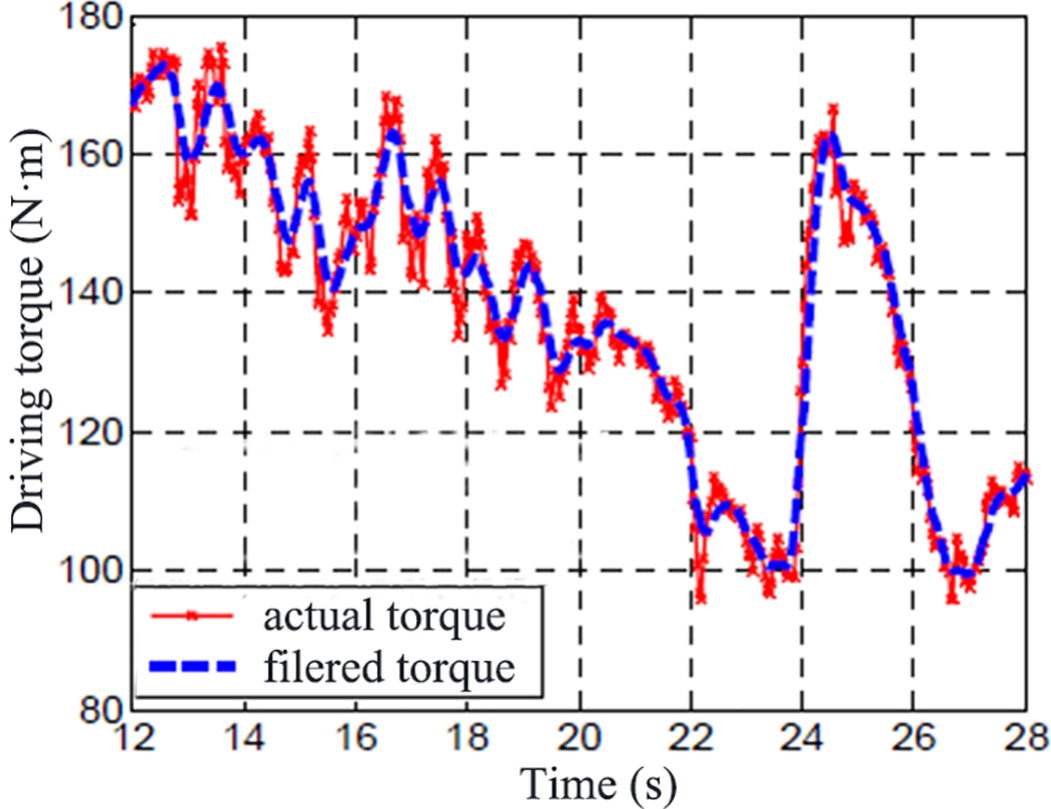
Original wheel torque and filtered.

Fig 15 shows the original wheel speed and filtered wheel speed. Fig 16 shows the original wheel torque and filtered wheel torque. Fig 15 and Fig 16 shows the test result of driving condition with an input of high throttle percentage on icy road. It can be seen the filtered signals reflect the changes of the original data with a time delay.

**Fig 17:**
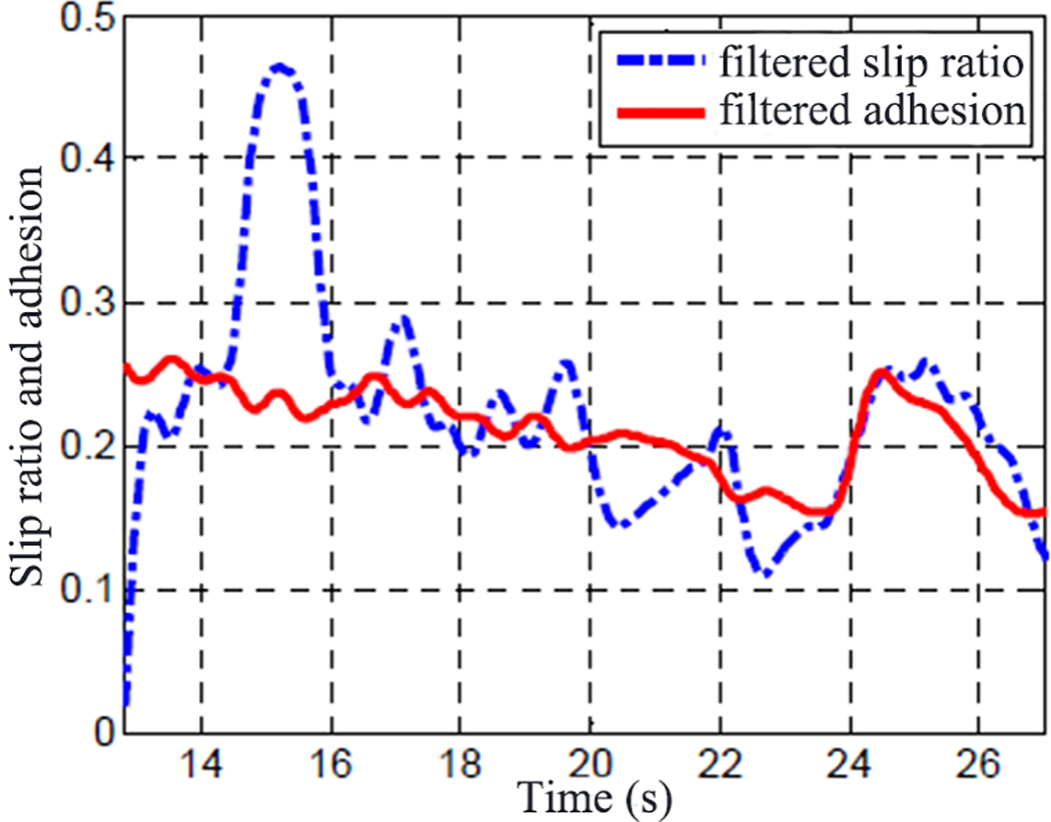
Slip ratio and adhesion coefficient.

Fig 17 shows the slip ratio and the adhesion coefficient, which are achieved by calculating the data using Eq 4. It can be seen that the driving wheel began to skid with a large slip ratio from the non-skid state (14 s ~ 16 s), and then the wheel enters into an oscillatory state between non-skid and skid (after 16 s).

The adhesion coefficient maintains a larger value at the rising stage of the slip ratio, and the adhesion coefficient began to decrease when the slip ratio reaches a certain value. A higher slip ratio appears between 14s-16s, the adhesion coefficient shows a "bottom" trend during the period. When the slip ratio is reduced, the adhesion coefficient begins to rise again, and then the adhesion coefficient becomes smaller but the slip ratio also decreases at the same time. Another reason the slip ratio decreased with the adhesion coefficient is the ice-snow composite road, the road becomes more slippery because of the skid of the wheel, and the peak adhesion coefficient is decreased. At 24 s, the sudden increase in adhesion coefficient which is output by the identifier is caused by the road with a high adhesion coefficient onto which the car is running. The corresponding changes of the torque curve in Fig 15 also illustrates this point. Thus, the calculated adhesion coefficient is realistic.

**Fig 18:**
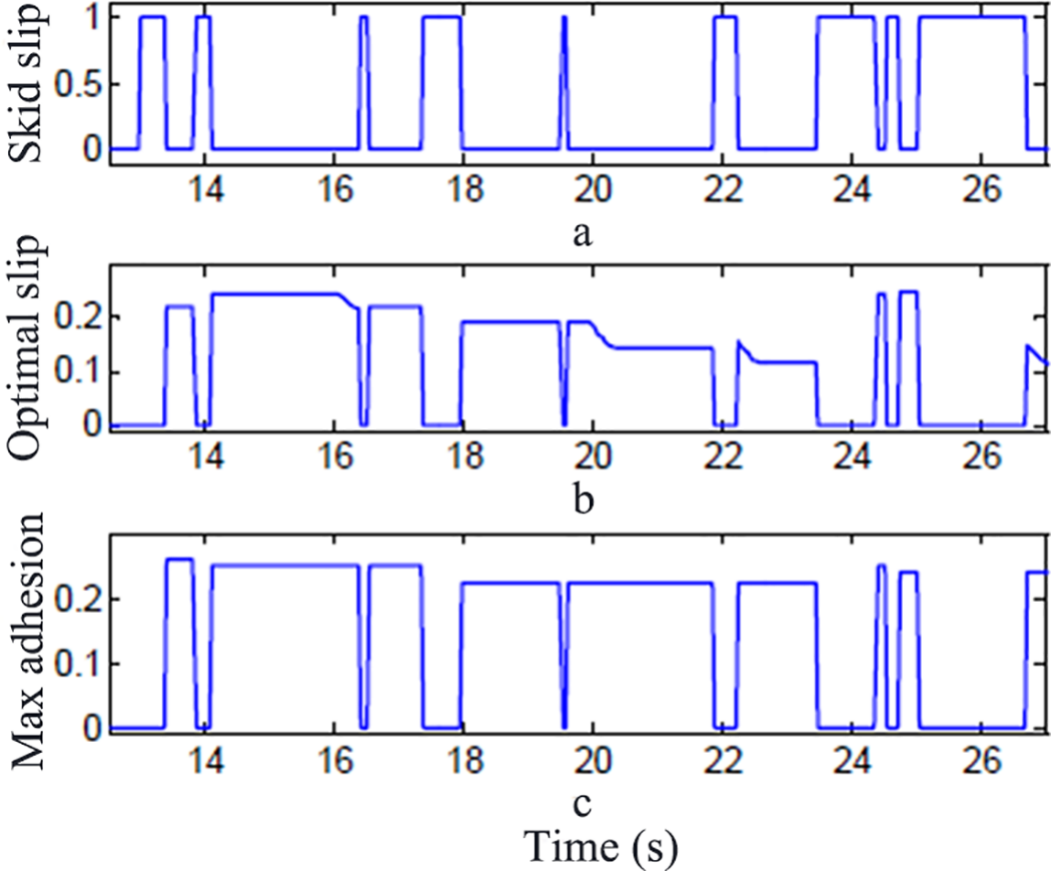
Output by road identifier.

Fig 16 shows the output of the wheel movement by the presented road identifier. Fig 18A shows the indicator of the wheel skid state where "1" stands for the state of no skid, and "0" stands for the state of skid. Fig 18B shows the optimum slip ratio output by the identifier when in the skid state. Fig 18C shows the peak adhesion coefficient when in the skid state. When the wheels do not skid, the optimum slip ratio and the peak adhesion coefficient output is 0.

From the above figures, the identifier can accurately identify the skid state according to the changes of the road adhesion coefficient and the slip ratio and output the optimum slip ratio and the peak adhesion coefficient. The inconstant skid states output by the identifier is caused by the fluctuation of the adhesion coefficient and the slip ratio.

## 5. Conclusion

Traction control system (TCS) for in-wheel-motor (IWM) configuration electric vehicles (EV) has advantages, the performance of the control system, comparing with the traditional method, the method proposed here significantly decreases the time and the remarkable improve recognition ratio, and the feedback slip control appear to be better than with a conventional powertrain.

This paper attempts to identify in real-time the optimal longitudinal slip ratio and the corresponding maximum available road friction when drive torque exceeds or is close to the available road friction. It proposes the identification of the peak longitudinal force based on the sign change of the derivative of calculated longitudinal force. Although the algorithm is relatively simple, but it can enhance the speed and computing efficiency. Furthermore, the non-skid minimum wheel velocity is used to estimate the chassis velocity, which are key parameters of the TCS. The estimated slip ratio ensures that the vehicle stay within reasonable ranges. Finally, the simulation and experimental results show that the proposed algorithm can identify the variation of road surface adhesion characteristics without wheel and chassis velocity sensors. The traction control system and dynamic control system for the development of high performance and low cost electric wheel vehicle are presented.

## Supporting information

S1 File Zip. (ZIP)
